# Supplementary material for: Multiomics Approach Captures Hepatic Metabolic Network Altered by Chronic Ethanol Administration
Source: Biology (Basel). 2022 Dec 23;12(1):28. doi: 10.3390/biology12010028 (PMC9855439; doi:10.3390/biology12010028)
Supplement: Supplementary file 1 [file biology-12-00028-s001.zip › biology-2022400-supplementary.pdf]

Supplemental Information

For

Multiomics Revealed the Hepatic Metabolic Pathways Altered by Chronic Ethanol Administration

Isin Tuna Sakallioglu,[1] Bridget Tripp[2], Jacy Kubik,[3,4] Carol A. Casey,[3,4] Paul Thomes,[3,4*] and Robert Powers[1,5*]

[1]*Department of Chemistry, University of Nebraska-Lincoln, Lincoln NE 68588-0304*

[2]*Complex Biosystems, University of Nebraska-Lincoln, Lincoln, NE 68588-0619*

[3]*Departments of Internal Medicine and* [4]*Biochemistry and Molecular Biology, University of Nebraska Medical Center, Omaha, NE 68198-5870*

[5]*Nebraska Center for Integrated Biomolecular Communication, University of Nebraska-Lincoln, Lincoln NE 68588-0304*

Contents:

Material and Methods

Figures:

Figure S1. Bar plot of the number of PubMed papers published in the last seven years that used metabolomics, lipidomics, and proteomics to investigate alcoholic liver disease.

Figure S2. A distribution of feature intensities from the combined omics dataset before (*left*) and after (*right*) the data was normalized and scaled. The resulting bell-shaped curve demonstrates the good quality of the normalization scheme and the corresponding reliability of the combined omics dataset.

Figure S3. Bar plots of A) the relative amounts of *Ugt-1A1* mRNA (*p*-value < 0.05, F.C. 1.8 higher in control) and B) UGT enzymatic activity detected from hepatocytes cells isolated from control and EtOH-fed rat livers.

Figure S4. Boxplot of the relative amounts of the *Cpt1A* protein detected in hepatocytes cells isolated from control and EtOH-fed rat livers (*p*-value < 0.01, F.C. 3.3 times higher in ethanol).

Tables:

Table S1. Summary of quality and validation metrics for the PCA and OPLS-DA models.

Table S2. Summary of the top-enriched metabolites from the LC-MS and NMR datasets.

Table S3. Summary of the top-enriched lipids from the LC-MS dataset.

Table S4. Enriched pathway analysis of proteomics dataset using KEGG.

Table S5. Enriched pathway analysis of proteomics dataset using Wikipathways.

References

Methods and Materials

Animal model of alcoholic liver disease (ALD). Alcohol consumption in humans begins with the accumulation of fat in the liver (fatty liver). Continuous use or abuse of alcohol increases the risk of disease progression from fatty liver to fibrosis, cirrhosis, and eventually liver cancer or death. There are many different alcohol consumption patterns in humans, such as binge drinking, acute, or chronic alcohol consumption. Both the amount of alcohol consumption and the drinking pattern influence the pathological outcomes.

The ALD animal model employed in our study consisted of Wister rats fed a standard Lieber-DeCarli diet supplemented with 36% EtOH for 5-8 weeks. Thus, this ALD animal model mimics a chronic alcohol consumption pattern. This model leads to the development of fatty liver and moderate liver injury. Since the focus of our investigation is delineating the changes in hepatic metabolic network during the development of alcohol induced fatty liver, we chose to use this alcohol feeding animal model.

Hepatocyte cell samples. The Wistar rats in this experiment were received from Charles River Labs. Approximately 6 replicates of $10x10^6$ hepatocyte cells were harvested from healthy control rats (n=7) and rats dosed with ethanol (n=6). The cells were counted and then stored as pellets at -80°C. The samples were randomized throughout the extraction, sample preparation and data collection protocols.

Aqueous metabolites extraction. The cells were thawed and then washed by adding 1 mL of Nanopure water to each cell pellet followed by pipet mixing. The cells were then centrifuged at

5000 g for 10 minutes at 4°C. The cell wash was discarded. A quality control (QC) sample was prepared before metabolite extraction. 20 μL was removed from each hepatocyte cell biological replicate sample and combined to produce the single QC sample. The same extraction procedure was applied to both the QC and hepatocyte cell samples. Cells were resuspended in 1 mL of 80% methanol and submitted to mechanical lysis with zirconia beads in a FastPrep® homogenizer (15 s at 1200 rpm followed by 30 s in an ice bath, the cycle was repeated three times). The lysed sample was then centrifuged at 20,000 g for 20 minutes at 4°C. The supernatant was collected and 1 mL of 50% methanol in Nanopure water was added to each cell pellet and vortexed for 10 seconds. The sample was centrifuged, the supernatant was collected and combined with the first extract. The sample was split 90:10 to prepare both an NMR and mass spectrometry (MS) sample, respectively. Each sample was transferred to a centrifugal evaporator and then lyophilized to dryness. In this regard, an NMR and MS sample was both prepared from each individual hepatocyte cell sample.

Organic lipid extraction. Upon completion of the aqueous extraction, 4 mL of a 2:1:1 chloroform:methanol:water mixture was added to each cell pellet in a glass vial. The pellet was vortexed for 30 seconds and then centrifuged at 20,000 g for 20 minutes at 4°C. The organic layer was collected, and the process was repeated two additional times. The three organic extracts were combined and then dried with a rotary evaporator. The organic extraction was only used to prepare a MS sample.

Preparation of NMR aqueous metabolome samples. After lyophilization, each dried NMR aqueous sample was resuspended in 0.3 mL of a 50 mM potassium phosphate buffer at pH 7.2

(uncorrected) in “100%” D_2O containing 50 μM TMSP-D_4 as a chemical shift reference. The sample was transferred to a 3 mm NMR tube for data collection.

Preparation of liquid-chromatography-mass spectrometry (LC-MS) aqueous metabolome samples. After lyophilization, each dried MS aqueous extract was resuspended in 100 uL of Nanopure water containing 0.1% of formic acid.

Preparation of LC-MS organic metabolome samples. Each dried MS organic extract was resuspended in 100 uL of isopropanol containing 0.1% formic acid.

One-dimensional (1D) ^{1}H NMR data acquisition. 1D ^{1}H NMR spectra were collected on a Bruker Avance III-HD 700 MHz spectrometer equipped with a quadruple resonance QCI-P cryoprobe (^{1}H, ^{13}C, ^{15}N, ^{31}P) with z-axis gradients. A Bruker SampleJet sample changer with IconNMR and an auto tune and match (ATM) system were used to automate the NMR data collection. The 1D ^{1}H NMR spectra were collected at 298K with 32K data points, a spectral width of 11 ppm, 64 scans and 4 dummy scans. The spectra were collected using excitation sculpting to remove the solvent and maintain a flat baseline.[1]

LC-MS metabolomics data acquisition. LC-MS Metabolomics was performed on a Waters Acquity UPLC system coupled to a Xevo G2-XS Q-TOF (Waters MS Technologies, Manchester, UK) equipped with an electrospray ionization (ESI) source operating in positive ionization mode. Two mobile phases were prepared, mobile phase A consisted of 0.1% formic acid in water and mobile phase B was 0.1% formic acid in acetonitrile. The metabolites were separated with a Waters

high strength silica (HSS-T3, 1.0 mm × 50 mm, 1.8 µm) column with a 31-minute linear gradient from 0.1% to 85% B. Column and autosampler temperature were set to 40°C and 5°C, respectively. The flow rate was set to 95 µL/min. The ionization source condition was set as follows: capillary voltage of 3.2 kV, sampling cone voltage of 40 V and source offset of 80 V. The source temperature was set to 120°C and the desolvation temperature was set to 500°C. The cone and desolvation gas flows were set to 50 and 800 L/h, respectively. Data acquisition was in MS^E mode, which simultaneously records exact mass precursor and fragment ion information. MS^E was performed with a low collision energy of 4 eV and the high collision energy ramped from 15 to 50 eV. The data was collected using an *m/z* range of 50 to1,200 with a scan time of 0.05 seconds. The data were acquired using an independent reference lock mass via the LockSpray interface to ensure accuracy and reproducibility during the MS analysis. Leucine Enkephalin was used as the reference compound ($[M+H]^+$ = 556.2771).

LC-MS lipidomics data acquisition. LC-MS Lipidomics was performed on a Waters Acquity UPLC system coupled to a Xevo G2-XS Q-TOF (Waters MS Technologies, Manchester, UK) equipped with an ESI source operating in either positive or negative ionization mode. Two mobile phases A was composed of acetonitrile/water (60:40, *v/v*) mixture containing ammonium formate (10 mM, pH 6.2) and formic acid (0.1%). Mobile phase B was composed of an isopropanol/acetonitrile mixture (90:10, *v/v*) containing ammonium formate (10 mM, pH 6.2) and formic acid (0.1%). The lipids were separated with a Waters C18 column Charged Surface Hybrid (CSH, 1.0 × 50 mm, 1.7 μm) column with a 20-minute gradient in 90% mobile phase B. Column and autosampler temperature were set to 40°C and 5°C, respectively. The flow rate was set to 50 µL/min. The ionization source condition was set as follows: capillary voltage of 3.2 kV, sampling

cone voltage of 40 V and source offset of 80 V. The source temperature was set to 120°C and the desolvation temperature was set to 500°C. The cone and desolvation gas flows were set to 50 and 800 L/h, respectively. Tandem mass spectrometry in data independent acquisition mode was performed with a low collision energy of 4 eV and the high collision energy was ramped from 15 to 50 eV. The data was collected using an *m/z* range of 50 to1,200 with a scan time of 0.05 seconds. The data were acquired using an independent reference lock mass via the LockSpray interface to ensure accuracy and reproducibility during the MS analysis. Leucine Enkephalin was used as the reference compound ($[M+H]^+$ = 556.2771). Splash® Lipidomics ® Mass Spectrometry Standard (Avanti polar lipids, Inc.) was used as quality-control standard before collecting the experimental lipidomics samples.

NMR data processing. The 1D ^{1}H NMR spectra were processed with our MVAPACK metabolomics toolkit (http://bionmr.unl.edu/mvapack.php)[2] to generate a data matrix and perform multivariate and univariate statistical analysis. The spectra were processed with a 1.0 Hz exponential apodization function, a single round of zero-filling, and a Fourier transformation. Spectra was referenced and aligned to TMSP-D_4.[2] Solvent signals and noise regions were excluded, and the spectra were either aligned using the Icoshift peak alignment algorithm or binned using an adaptive intelligent binning algorithm. The 1D ^{1}H NMR data matrix was pareto scaled and normalized with the median in order to generate a normal gaussian distribution using MetaboAnalyst 5.0.[3] The principal component analysis orthogonal projection to latent structures discriminant analysis (OPLS-DA) models were generated using MVAPACK.[2] The OPLS-DA models was validated using permutation testing with n = 1000, to yield associated R^2 , Q^2, and *p*-

values. Statistically significant features were selected using the generated data matrix from the OPLS-DA model.

NMR metabolite identification. Discriminatory features were identified and quantified using Chenomx NMR Suite 8.3 (Chenomx, Edmonton, Canada). The Chenomx-Processor module was first used to baseline and phase correct the 1D ^{1}H NMR spectra. The NMR spectra were then assigned and the corresponding areas under the curve (AUC) were calculated by computer assisted manual batch fitting. The AUC and metabolite assignments were exported to an Excel file. Student's t-test was used to measure pair-wise statistical significance (p-value < 0.05), which was then corrected for a false-discover rate (FDR) with the Benjamini-Hochberg multiple hypothesis correction. The AUC fold changes were calculated by comparing the average AUC of the ethanol treatment group to the average AUC of the control groups.

LC-MS metabolomics data peak picking. The LC-MS datasets were imported to Progenesis® QI metabolomics software (version 2.4, Nonlinear Dynamics, Newcastle, UK). The chromatographic alignment, peak picking, and data normalization (with normalization to all features) were performed in an automatic manner with quality control (QC) runs as references. Features were automatically deconvoluted for isotopes and adducts, and then associated with a retention time and a *m/z* value. For positive ionization mode, adducts were assigned to either $[M+H]^+$, $[M+Na]^+$, $[M+NH_4]^+$, $[M+2H]^+$, $[M-H+2Na]^+$, $[M–H_2O+H]^+$, $[M+H-2H2O]^+$, or $[M+K]^+$. Features were selected based on coefficients of variation (CVs) relative to QC samples where features with CVs over 30% were eliminated.

LC-MS lipidomics data peak picking. The LC-MS datasets were imported to Progenesis® QI metabolomics software (version 2.4, Nonlinear Dynamics, Newcastle, UK). The chromatographic alignment, peak picking, and data normalization (with normalization to all features) were performed in an automatic manner with quality control (QC) runs as references. Features were automatically deconvoluted for isotopes and adducts, and then associated with a retention time and a *m/z* value. For positive ionization mode, adducts were assigned to either as $[M+H]^+$, $[M+Na]^+$, $[M+NH_4]^+$, $[M+2H]^+$, $[M+H+Na]^{2+}$, $[M–H_2O+H]^+$, or $[M+K]^+$. Features were selected based on coefficients of variation (CVs) relative to QC samples where features with CVs over 30% were eliminated.

Data preprocessing and post-processing. A feature was considered present if it was measured in at least 50% of the samples. Features deemed absent were omitted while signal imputation was performed on the remaining features using the k-nearest neighbor (KNN) method.[4] All features underwent a log2 transformation. LC-MS metabolic features were normalized using cubic splines.[5] LC-MS lipid and metabolic features were normalized using probabilistic quotient normalization (PQN).[6] Signal drift was corrected using a quality control-based machine learning algorithm: random forest signal correction (QC-RFSC).[7] All preprocessing was performed using NOREVA 2.0 online service.[8]

Combined dataset post-processing. The post-processed data matrices were concatenated into a single matrix using only those samples found across all three data types. Furthermore, the combined matrix was scaled using the auto-scaling method in MetaboAnalyst 5.0 to derive a

normalized Gaussian curve. The multivariate and univariate statistical analysis was performed using MetaboAnalyst 5.0.[25]

LC-MS statistical analysis. The multivariate and univariate statistical analysis was performed using MetaboAnalyst 5.0.[25] PCA and OPLS-DA models were calculated after pareto scaling for outlier detection and comparative analysis between groups. The loading plots and S-plots were generated from the OPLS-DA model and were employed to visualize the relative importance of the differential variables and to acquire a list of peak indices. Metabolites were considered differential when presenting a minimum fold change ≥ 1.5 and/or ANOVA p-value ≤ 0.05. The qualified filtered variables were further submitted to identification.

Metabolite and lipid identification from the MS dataset. The features and corresponding peak area for the metabolite were subjected to percent standard deviation calculation (30% < percent standard deviation), Student's t-test (p-value < 0.05) with multiple hypothesis correction (Benjamini-Hochberg), and fold change calculation. The differential features were identified using the Progenesis QI metabolomics software by searching *m/z* values and retention times against reference entries in the Human Metabolome Database using accurate mass measurements, 5 ppm mass error.[9,10] The fragmentation information was also used to increase the confidence in lipid and metabolite identification, as was stated in the paper by Crook and Chatterjee *et al.*[11,12]

Metabolite Pathway and Network analysis. After univariate statistical analysis and compound identification, the statistically significant features from both the LC-MS and NMR datasets were subjected to network analysis using MetaboAnalyst 5.0.[3,13] Significantly perturbed metabolite

pathways were identified using the Pathway Analysis module in MetaboAnalyst 5.0 by selecting all the metabolite names identified by NMR and MS metabolomics data analysis.

Proteomics Extraction. 50 µg of protein per sample was obtained from each of the five biological replicates per group. Detergent was removed by a chloroform/methanol extraction. The protein pellet was then re-suspended in 100 mM ammonium bicarbonate and digested overnight at 37°C with MS-grade trypsin (Pierce). Sample reduction occurred with 10 mM DTT at 56°C for 30 mins and alkylation was accomplished using 50 mM iodoacetamide at RT for 25 mins. Peptides were cleaned with PepClean C18 spin columns (Thermo) and were re-suspended in 2% acetonitrile (ACN) and 0.1% formic acid (FA).

Proteomics LC-MS data acquisition. 500 ng of each sample was loaded onto trap column Acclaim PepMap 100 75 µm x 2 cm C18 LC Columns (Thermo Scientific™) at flow rate of 4 µl/min then separated with a Thermo RSLC Ultimate 3000 (Thermo Scientific™) on a Thermo Easy-Spray PepMap RSLC C18 75 µm x 50 cm C-8 2 µm column (Thermo Scientific™) with a step gradient of 4–25% solvent B (0.1% FA in 80 % ACN) from 10-100 min and 25–45% solvent B for 100–130 min at 300 nL/min and 50ºC with a 155 min total run time. Eluted peptides were analyzed by a Thermo Orbitrap Fusion Lumos Tribrid (Thermo Scientific™) mass spectrometer in a data dependent acquisition mode. A survey full scan MS (from *m/z* 350–1800) was acquired in the Orbitrap with a resolution of 120,000. The AGC target for MS1 was set as 4×10^5 and ion filling time set as 100 ms. The most intense ions with charge state 2-6 were isolated in 3 s cycle and fragmented using HCD fragmentation with 35% normalized collision energy and detected at a mass resolution of 30,000 at 200 *m/z*. The AGC target for MS/MS was set to 5×10^4, ion filling

time was set to 60 ms, and dynamic exclusion was set for 30 s with a 10 ppm mass window. Each sample was run in duplicate.

Protein Identification and Statistics. Protein identification was performed by searching MS/MS data against the Swiss-Prot rat protein database downloaded on July 2020 using the in house PEAKS X + DB search engine. The search was set up for full tryptic peptides with a maximum of two missed cleavage sites. Acetylation of protein N-terminus and oxidized methionine were included as variable modifications and carbamidomethylation of cysteine was set as fixed modification. The precursor mass tolerance threshold was set to 10 ppm and a maximum fragment mass error was set to 0.02 Da. The significance threshold of the ion score was calculated based on a false discovery rate of ≤ 1%. Quantitative data analysis was performed using progenesis QI proteomics 4.2 (Nonlinear Dynamics). Statistical analysis was performed using ANOVA and The Benjamini-Hochberg method was used to adjust *p*-values for multiple-testing false discovery rate. An FDR adjusted *p*-value ≤ 0.05 was considered as significant.

Protein Network Analysis. A network analysis of differentially expressed proteins was performed using Cytoscape v 3.9.0 with the ClueGo v 2.5.8 plugin.[14,15]

Quantitative Real Time-Polymerase Chain Reaction measurement of *Ugt-1a1.* Total RNA was isolated from hepatocytes cells using the NucleoSpinRNA kit (Macherey Nagel, Düren, Germany) according to the manufacturer's recommendations. Total RNA concentration was quantified using a NanoDrop spectrophotometer and stored at −80°C until further use. 500 ng of the extracted RNA was reverse transcribed using PrimeScript RT Master Mix (Takara, Saint-

Germain-en-Laye, France) according to the manufacturer's recommendations. The transcribed cDNA was then used to determine the regulation of *Ugt-1a1* in the hepatocytes by qPCR using SYBR Premix Ex Taq II (Takara). *Ugt-1a1* primers were selected based on the primer sequences reported by Kutsukake *et al*.[16] For the reaction, 25 ng of cDNA was used as a template and added to 1 × SYBR Premix Ex Taq II together with each respective primer pair at a final concentration of 0.6 µM. The qRT-PCR cycling protocol was set to 45 cycles (10 s at 94°C, 10 s at 60°C, and 10 s at 72°C), followed by a melting curve analysis. Transcript levels were calculated by relative quantification using the GapDH as an internal reference and the normalized abundances were plotted. The qRT-PCR was carried out with the Light Cycler R480 (Roche, Basel, Switzerland).

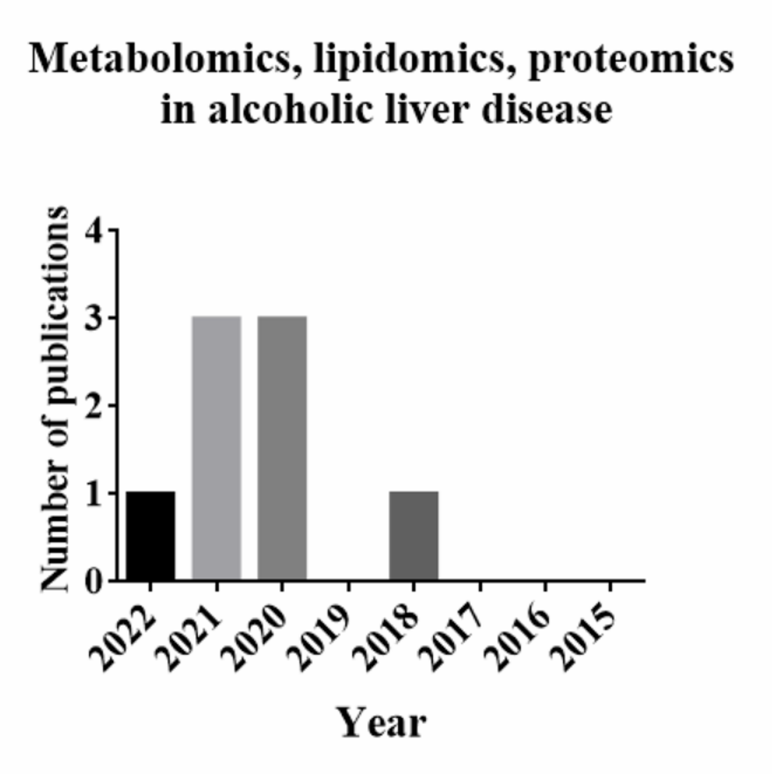

Figure S1. Bar plot of the number of PubMed papers published in the last seven years that used metabolomics, lipidomics, and proteomics to investigate alcoholic liver disease.

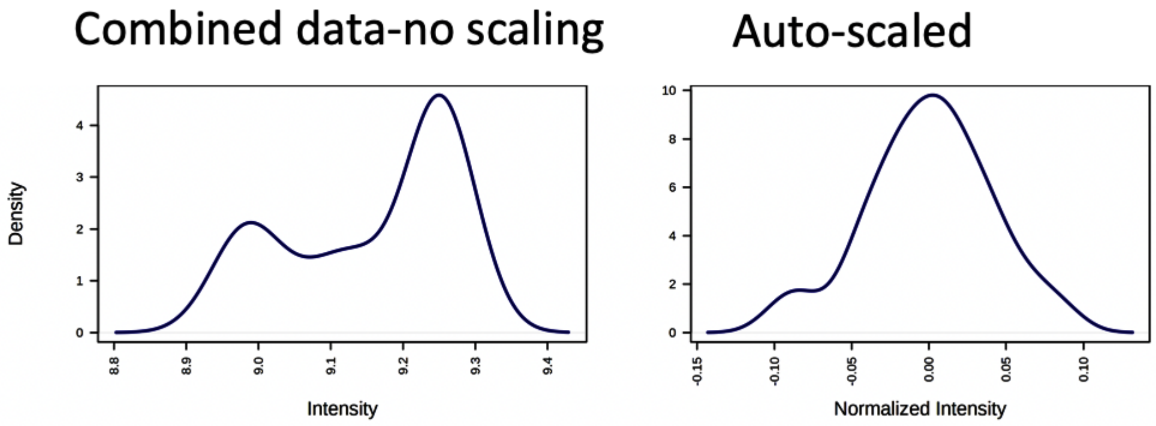

Figure S2. A distribution of feature intensities from the combined omics dataset before (*left*) and after (*right*) the data was normalized and scaled. The resulting bell-shaped curve demonstrates the good quality of the normalization scheme and the corresponding reliability of the combined omics dataset.

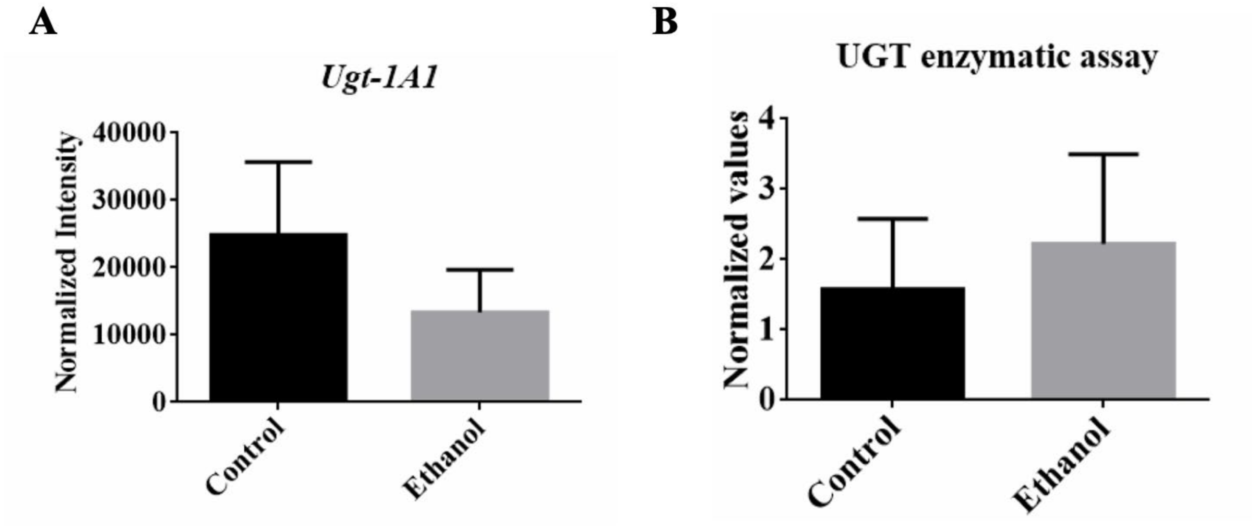

Figure S3. Bar plots of A) the relative amounts of *Ugt-1A1* mRNA (*p*-value < 0.05, F.C. 1.8 higher in control) and B) UGT enzymatic activity detected from hepatocytes cells isolated from control and EtOH-fed rat livers

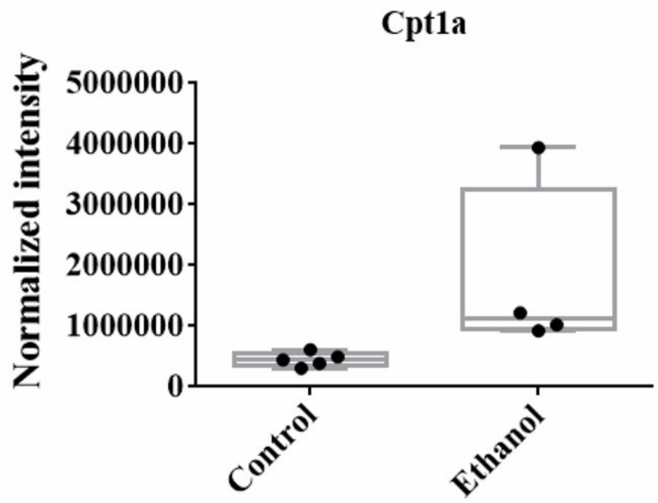

Figure S4. Boxplot of the relative amounts of the *Cpt1A* protein detected in hepatocytes cells isolated from control and EtOH-fed rat livers (p-value < 0.01, F.C. 3.3 times higher in ethanol).

Table S1. Summary of quality and validation metrics for the PCA and OPLS-DA models.

Omics dataset	**PCA**[1]	***Validation metrics***[2]	**OPLS-DA**[3]	***Validation metric***[2]	***p-value***[4]
Metabolomics NMR	$\mathbf{R^2}$	0.507	$\mathbf{R^2}$	0.970	0.009
	$\mathbf{Q^2}$	0.451	$\mathbf{Q^2}$	0.945	0.006
Metabolomics MS	$\mathbf{R^2}$	0.791	$\mathbf{R^2}$	0.996	0.003
	$\mathbf{Q^2}$	0.775	$\mathbf{Q^2}$	0.994	0.003
Lipidomics MS	$\mathbf{R^2}$	0.875	$\mathbf{R^2}$	0.997	0.001
	$\mathbf{Q^2}$	0.857	$\mathbf{Q^2}$	0.995	0.001
Combined *NMR-MS Metabolomics-Lipidomics*	$\mathbf{R^2}$	0.755	$\mathbf{R^2}$	0.996	0.001
	$\mathbf{Q^2}$	0.734	$\mathbf{Q^2}$	0.994	0.001

[1]Principal component analysis
[2]R^2 provides a measure of model fit to the original data, and Q^2 provides a measure of internal consistency between the original and model predicted data.
[3]Orthogonal projection to latent structures
[4]*p*-values from a cross-permutation analysis with n=1000

Table S2. Summary of the top-enriched metabolites from the LC-MS and NMR datasets.

Metabolite class	Metabolite name	VIP[1]	*p*-value[2]	FDR *p*-value[3]	F.C.[4]	Average Control[5]	Average Ethanol[5]	MWW *p*-value[6]
**Glucuronides**	Dehydroisoandrosterone-glucuronide	1.08	$3.77x10^{-42}$	$4.87x10^{-41}$	0.04	3324.2	132.9	$6.69x10^{-14}$
	Estriol-glucuronide	0.89	$7.35x10^{-17}$	$7.72x10^{-17}$	2.04	225.8	459.6	$3.07x10^{-16}$
	Dehydrotestosterone glucuronide	0.91	$5.88x10^{-18}$	$6.50x10^{-18}$	0.25	430.4	107.9	$2.70x10^{-12}$
	Hydroxy-octadec-enoate-glucuronide	1.02	$2.03x10^{-29}$	$4.05x10^{-29}$	0.15	248.2	36.6	$7.67x10^{-14}$
	Hydroxyandrosterone-glucuronide	0.98	$6.78x10^{-22}$	$8.63x10^{-22}$	0.19	604.7	116.16	$3.56x10^{-17}$
	alpha-CEHC glucuronide	0.78	$3.88x10^{-12}$	$3.88x10^{-12}$	2.67	633.7	1691.7	$3.82x10^{-15}$
	Benzoyl glucuronide (Benzoic acid)	1.09	$4.81x10^{-42}$	$4.87x10^{-41}$	58.62	4347.7	254848.3	$2.51x10^{-22}$
	Cholestane-tetrol-glucuronide	0.93	$4.02x10^{-20}$	$4.97x10^{-20}$	0.29	1115.5	320.3	$2.40x10^{-22}$
	Cholic acid glucuronide	0.97	$6.37x10^{-23}$	$8.63x10^{-23}$	2.66	442.1	1174.1	$8.00x10^{-22}$
	Phenethylamine glucuronide	1.07	$3.74x10^{-39}$	$1.74x10^{-38}$	0.05	212.9	10.2	$5.69x10^{-14}$
	Tyramine glucuronide	1.08	$1.04x10^{-41}$	$7.27x10^{-41}$	43.41	2.2	96.1	$1.82x10^{-14}$
**Amino acids**	Sarcosine	0.93	$3.57x10^{-8}$	$4.91x10^{-8}$	2.67	0.5	1.4	$9.80x10^{-06}$
	Betaine	1.48	$1.94x10^{-62}$	$3.21x10^{-61}$	3.26	1.9	6.2	$5.25x10^{-12}$
	Glycine	1.29	$4.18x10^{-20}$	$1.53x10^{-19}$	4.66	1.2	5.6	$7.15x10^{-10}$
	Alanine	1.47	$2.77x10^{-47}$	$3.05x10^{-46}$	3.38	6.5	22.0	$5.23x10^{-12}$
	Lysine	0.98	$1.68x10^{-9}$	$2.77x10^{-9}$	2.41	5.5	13.2	$1.34x10^{-07}$
	Valine	1.31	$6.63x10^{-23}$	$3.13x10^{-22}$	2.07	5.9	12.2	$5.17x10^{-12}$
**Leukotrienes**	Oxo-dihydroxy-leukotriene B4	1.09	$1.36x10^{-41}$	$8.15x10^{-41}$	8.89	42.2	375.2	$2.31x10^{-22}$
	COOH-leukotriene E4	0.8	$3.32x10^{-13}$	$3.41x10^{-13}$	2.76	302.3	835.7	$8.00x10^{-12}$
	Dihydroxyleukotriene B4	1.08	$1.34x10^{-36}$	$4.68x10^{-36}$	0.33	1463.7	488.3	$5.14x10^{-14}$
	Leukotriene E4	0.98	$7.08x10^{-23}$	$9.30x10^{-23}$	0.41	436.7	177.7	$8.00x10^{-22}$
**Monosaccharides**	Ribose	1.07	$2.85x10^{-11}$	$5.23x10^{-11}$	0.33	0.9	0.3	$7.98x10^{-08}$
	Xylose	0.97	$4.76x10^{-9}$	$7.14x10^{-9}$	2.1	7.5	15.7	$1.15x10^{-08}$
	Xylulose	0.77	$7.05x10^{-6}$	$8.31x10^{-6}$	2.16	5.1	11.0	$1.81x10^{-4}$
**Cholines**	Choline	0.88	$2.28x10^{-7}$	$3.01x10^{-7}$	3.09	1.1	3.3	$1.24x10^{-5}$
	Phosphorylcholine	0.35	$6.97x10^{-2}$	$6.97x10^{-2}$	2.08	9.7	20.2	$9x10^{-2}$
**Monoacylglycerophosphoethanolamines**	LysoPE(16:0)	1.01	$1.04x10^{-28}$	$1.82x10^{-28}$	5.66	36.5	206.8	$9.11x10^{-14}$
	LysoPE(20:5)	0.94	$5.88x10^{-20}$	$7.05x10^{-20}$	0.42	734.1	307.0	$8.76x10^{-18}$
	LysoPE(22:4)	1.07	$5.80x10^{-42}$	$4.87x10^{-41}$	0.06	792.0	47.4	$5.61x10^{-14}$
**C18 steroids**	Oxoestrone	1.05	$6.55x10^{-34}$	$1.62x10^{-33}$	3.61	35.9	129.7	$5.73x10^{-14}$
	Hydroxyestrone	0.98	$6.19x10^{-24}$	$9.62x10^{-24}$	0.2	327.9	66.2	$5.72x10^{-14}$
	Hydroxyestradiol	1.04	$4.09x10^{-33}$	$9.53x10^{-33}$	0.14	220.4	30.1	$5.73x10^{-14}$
**TCA acids**	Citric acid	1.19	$1.65x10^{-15}$	$3.89x10^{-15}$	6.33	0.3	1.9	$4.18x10^{-11}$
	Succinic acid	1.37	$6.04x10^{-27}$	$3.32x10^{-26}$	2.75	0.4	1.1	$5.14x10^{-12}$

**Dicarboxylic acids**	Galactaric acid	1.23	$4.59x10^{-17}$	$1.17x10^{-16}$	3.29	0.7	2.3	$8.87x10^{-11}$
	Succinic acid	1.37	$6.04x10^{-27}$	$3.32x10^{-26}$	2.68	0.4	1.07	$5.14x10^{-12}$
	Undecanedioic acid	0.38	$3.12x10^{-2}$	$3.22x10^{-2}$	1.23	3.0	3.7	$4.12x10^{-02}$
**Fatty acyl glycosides**	Octanol glucoside	0.96	$2.71x10^{-23}$	$3.92x10^{-23}$	3.02	144.4	436.9	$8.00x10^{-22}$
	Hexanol arabinosylglucoside	0.96	$2.28x10^{-23}$	$3.41x10^{-23}$	0.27	226.6	61.2	$3.12x10^{-13}$
**Hydroxy Fatty Acids**	Hydroxyisovaleric acid	0.98	$2.10x10^{-9}$	$3.29x10^{-9}$	3.75	0.4	1.5	$1.58x10^{-6}$
	Hydroxymethylglutaric acid	1.03	$2.23x10^{-10}$	$3.87x10^{-10}$	3.63	0.8	2.9	$5.56x10^{-7}$
	Glycolic acid	0.42	$2.64x10^{-2}$	$2.81x10^{-2}$	1.59	1.7	2.7	$3.12x10^{-2}$

[1]VIP score - variable importance in projection score from OPLS-DA model
[2]*p*-value - Student's t-test *p*-value
[3]FDR *p*-value – FDR corrected *p*-value using the Benjamini-Hochberg method
[4]F.C. - fold change calculated using the average of integrated peak area from EtOH-feed samples divided by the average of the integrated peak area from control samples.
[5]Average feature intensity from the control or EtOH-feed samples
[6]Mann–Whitney U test or Wilcoxon Rank Sum Test *p*-value

Table S3. Summary of the top-enriched lipids from the LC-MS dataset.

Lipid class	Lipid name	VIP[1]	p-value[2]	FDR p-value[3]	F.C.[4]	Average Control[5]	Average Ethanol[5]	MWW *p*-value[6]
**Diacylglycerols**	DG(32:3)	1.1	$3.55x10^{-28}$	$7.96x10^{-28}$	5.08	34.0	172.4	$4.57x10^{-14}$
	DG(34:3)	0.81	$3.57x10^{-12}$	$4.20x10^{-12}$	0.47	2242.5	1061.9	$1.70x10^{-17}$
	DG(36:3)	0.11	$3.64x10^{-1}$	$3.64x10^{-1}$	0.04	31367.7	1210.2	$1.70x10^{-17}$
	DG(39:4)	1.01	$9.88x10^{-22}$	$1.37x10^{-21}$	0.3	831.8	252.5	$4.65x10^{-19}$
	DG(42:1)	1.11	$8.44x10^{-35}$	$6.61x10^{-34}$	7.75	470.8	3650.7	$1.11x10^{-13}$
	DG(42:2)	1.02	$2.09x10^{-24}$	$3.65x10^{-24}$	5.03	257.2	1294.6	$7.93x10^{-13}$
**Thia Fatty Acids**	Mercapto-octadecanoic acid	1.13	$7.43x10^{-46}$	$3.49x10^{-44}$	11.22	38.6	433.5	$4.87x10^{-14}$
**Diacylaminosugars**	UDP-hydroxymyristoyl-GlcNAc	1.03	$1.84x10^{-23}$	$3.09x10^{-23}$	0.15	1404.7	215.2	$1.70x10^{-17}$
**Diacylglycosylglycerophospholipids**	PC(17:0,20:4)	1.06	$2.89x10^{-23}$	$4.69x10^{-23}$	2.78	1239.6	3447.7	$2.06x10^{-21}$
**Other Eicosanoids**	HEDE	1.06	$5.58x10^{-25}$	$1.01x10^{-24}$	2.84	360.0	1021.9	$1.71x10^{-22}$
**Triacylglycerols and triradylcyglycerol**	TG(58:1)	1.01	$2.84x10^{-19}$	$3.71x10^{-19}$	6.24	5228.3	32606.9	$1.77x10^{-22}$
	TG(60:0)	1.12	$1.02x10^{-32}$	$4.35x10^{-32}$	8.77	1129.0	9900.5	$1.91 x10^{-22}$
	TG(50:0)	1.14	$8.18x10^{-39}$	$1.28x10^{-37}$	13.09	101.9	1334.1	$4.57 x10^{-14}$
	TG(59:2)	1.12	$3.92x10^{-33}$	$2.05x10^{-32}$	5.45	3081.8	16786.0	$5.14 x10^{-21}$
	TG(61:2)	1.14	$1.29x10^{-43}$	$3.02x10^{-42}$	12.19	375.6	4577.0	$5.08x10^{-14}$
	TG(60:4)	1.08	$1.77x10^{-31}$	$6.39x10^{-31}$	4.34	889.1	3859.1	$1.71x10^{-22}$
	TG(61:5)	1.09	$7.02x10^{-29}$	$1.94x10^{-28}$	4.8	444.8	2136.1	1.71x10-[22]
	TG(61:3)	1.12	$1.50x10^{-38}$	$1.76x10^{-37}$	5.56	615.4	3422.5	$5.09x10^{-14}$
	TG(62:1)	1.09	$3.82x10^{-34}$	$2.57x10^{-33}$	8.66	119.7	1036.0	$6.07x10^{-14}$
	TG(64:3)	1.13	$1.64x10^{-36}$	$1.54x10^{-35}$	10.31	1197.9	12351.3	$5.09x10^{-14}$
	TG(66:4)	1.07	$1.43x10^{-27}$	$3.05x10^{-27}$	5.53	259.0	1432.4	$4.94x10^{-14}$
	TG(68:5)	1.1	$3.19x10^{-28}$	$7.88x10^{-28}$	3.4	1210.4	4111.6	$8.78x10^{-14}$
**Hydroxysphinganines and sphinghanine**	Cer(44:1)	0.65	$1.50x10^{-6}$	$1.60x10^{-6}$	0.31	72501.2	22501.1	$2.52x10^{-17}$
	Cer(44:0(OH))	0.5	$4.12x10^{-4}$	$4.30x10^{-4}$	0.37	3109.2	1136.8	$8.53x10^{-13}$
**Phosphoserines and derivatives**	PS(39:0)	0.95	$1.15x10^{-19}$	$1.54x10^{-19}$	0.31	798.2	250.5	$2.99x10^{-12}$
	PS(44:3)	1.07	$9.41x10^{-33}$	$4.35x10^{-32}$	0.08	654.7	51.4	$3.57x10^{-13}$
	PS(O(42:4)	1.08	$4.36x10^{-30}$	$1.46x10^{-29}$	0.09	1111.8	99.2	$4.24x10^{-13}$
**Ceramide phosphoethanolamines**	PE-Cer(34:1)	1.07	$1.25x10^{-28}$	$3.27x10^{-28}$	0.11	421.4	44.8	$2.43x10^{-13}$
	PE-Cer(40:1(-OH))	0.99	$5.99x10^{-22}$	$8.80x10^{-22}$	0.35	2651.2	922.4	$1.70x10^{-17}$
**Carbocyclic Fatty Acids**	Phenyl heneicosanoic acid	0.77	$3.52x10^{-10}$	$3.93x10^{-10}$	0.49	1752.8	860.4	1.87x10-[12]
**phosphoethanolamines**	PE-NMe(32:0)	0.81	$9.59x10^{-12}$	$1.10x10^{-11}$	0.45	8898.2	3968.4	$4.78x10^{-13}$
	PE-NMe2(34:3)	1.07	$3.53x10^{-28}$	$7.96x10^{-28}$	0.12	3825.7	469.4	$3.95x10^{-18}$
	PE(P-36:3)	1.1	$1.02x10^{-29}$	$3.19x10^{-29}$	3.64	137.7	501.7	$9.48x10^{-14}$
**Stigmasterols**	Glc-Stigmasterol	0.98	$1.93x10^{-18}$	$2.45x10^{-18}$	2.24	880.2	1968.8	$9.88x10^{-19}$
**Prostaglandins**	PGF2alpha methyl ether	1.04	$6.80x10^{-22}$	$9.68x10^{-22}$	3.07	2552.0	7841.6	$1.15x10^{-20}$

**Cholesterol and derivatives**	Hydroxy -methyl-cholestene carboxylic acid	0.84	4.34x10^{-15}	5.37x10^{-15}	2.18	925.5	2016.9	3.11x10^{-16}
	Cholestane-tetrol	0.22	1.78x10^{-1}	1.82x10^{-1}	0.45	1253.2	567.6	2.04x10^{-6}
**Phosphocholine**	PC(37:4)	1.06	2.89x10^{-23}	4.69x10^{-23}	2.78	1239.6	3447.7	2.06x10^{-21}
**Unsaturated fatty acids**	tricosenoic acid	1.01	1.56x10^{-22}	2.45x10^{-22}	0.17	1940.9	338.3	1.71x10^{-22}

[1]VIP score - variable importance in projection score from OPLS-DA model
[2]*p*-value - Student's t-test *p*-value
[3]FDR *p*-value – FDR corrected *p*-value using the Benjamini-Hochberg method
[4]F.C. - fold change calculated using the average of integrated peak area from EtOH-feed samples divided by the average of the integrated peak area from control samples.
[5]Average feature intensity from the control or EtOH-feed samples
[6]Mann–Whitney U test or Wilcoxon Rank Sum Test *p*-value

Table S4. Enriched pathway analysis of proteomics dataset using KEGG.

Pathway name	Gene	*p*-value[1]	FDR *p*-value[3]	F.C.[3]
**Oxidative phosphorylation**	*LOC100911483;*	4.47x10^{-2}	4.77x10^{-2}	-2.81
	Cox6c2;	1.00x10^{-3}	1.34x10^{-2}	-2.6
	Ndufv2	1.08x10^{-3}	1.34x10^{-2}	-4.76
	Atp5f1d	4.21x10^{-3}	1.91x10^{-2}	-22.63
	Cox4i1	2.49x10^{-3}	1.73x10^{-2}	-3.09
	Cox5b	2.50x10^{-3}	1.73x10^{-2}	-5.16
	Ndufb10	3.70x10^{-3}	1.88x10^{-2}	-7.17
	Cox7a2l	4.35x10^{-3}	1.91x10^{-2}	-11.5
	Cox5a	4.44x10^{-3}	1.91x10^{-2}	-3.6
	Ndufa6	6.60x10^{-3}	2.28x10^{-2}	-3.88
	Ndufa10	7.80x10^{-3}	2.41x10^{-2}	-2.67
	Ndufs1	8.90x10^{-3}	2.54x10^{-2}	-3.3
	Ndufa2	1.37x10^{-2}	2.92x10^{-2}	-8.32
	Ndufs3	1.62x10^{-2}	3.13x10^{-2}	-2.01
	Ndufv3	1.76x10^{-2}	3.27x10^{-2}	-1.94
	Ndufs2	3.16x10^{-2}	4.16x10^{-2}	-2.62
**Metabolic pathways**	*Nt5e*	2.12x10^{-5}	3.91x10^{-3}	-4.46
	LOC100362216	4.47x10^{-2}	4.77x10^{-2}	-2.81
	Fah	4.05x10^{-4}	9.54x10^{-3}	-1.77
	Tm7sf2	4.15x10^{-4}	9.54x10^{-3}	3.51
	Rgn	4.99x10^{-4}	1.08x10^{-2}	-4.46
	Cyp2b1	7.02x10^{-4}	1.21x10^{-2}	6.98
	Cox6c2	1.00x10^{-3}	1.34x10^{-2}	-2.6
	Ndufv2	1.08x10^{-3}	1.34x10^{-2}	-4.76

	Atp5po	3.79x10^{-2}	4.52x10^{-2}	-3.22
	Sord	2.27x10^{-3}	1.73x10^{-2}	-2.56
	Cox4i1	2.49x10^{-3}	1.73x10^{-2}	-3.09
	Cox5b	2.50x10^{-3}	1.73x10^{-2}	-5.16
	Khk	2.65x10^{-3}	1.74x10^{-2}	-7.18
	Uroc1	3.23x10^{-3}	1.88x10^{-2}	-5.39
	Cyp2c13	3.67x10^{-3}	1.88x10^{-2}	-3.8
	Ndufb10	3.70x10^{-3}	1.88x10^{-2}	-7.17
	Cox5a	4.44x10^{-3}	1.91x10^{-2}	-3.6
	Enpp3	4.50x10^{-3}	1.91x10^{-2}	-2.11
	Plpp3	4.76x10^{-3}	1.95x10^{-2}	-3.24
	Ugt2b1	4.77x10^{-3}	1.95x10^{-2}	2.99
	Rdh2	5.50x10^{-3}	2.09x10^{-2}	-2.37
	Pc	5.62x10^{-3}	2.09x10^{-2}	-2.12
	Sqle	5.83x10^{-3}	2.14x10^{-2}	2.96
	Ndufa6	6.60x10^{-3}	2.28x10^{-2}	-3.88
	Ndufa10	7.80x10^{-3}	2.41x10^{-2}	-2.67
	Ndufs1	8.90x10^{-3}	2.54x10^{-2}	-3.3
	Eno1	9.33x10^{-3}	2.54x10^{-2}	-3.43
	Eno3	1.31x10^{-2}	2.83x10^{-2}	2.8
	Ndufa2	1.37x10^{-2}	2.92x10^{-2}	-8.32
	Cyp3a2	1.43x10^{-2}	3.00x10^{-2}	-4.03
	Aco2	1.55x10^{-2}	3.12x10^{-2}	-2.93
	Ndufs3	1.62x10^{-2}	3.13x10^{-2}	-2.01
	Cyp4a10	1.66x10^{-2}	3.13x10^{-2}	2.2
	Nsdhl	1.66x10^{-2}	3.13x10^{-2}	2.29
	Ndufv3	1.76x10^{-2}	3.27x10^{-2}	-1.94
	Comt	2.05x10^{-2}	3.47x10^{-2}	-4.3
	Dhcr7	2.38x10^{-2}	3.67x10^{-2}	2.22
	Uox	2.96x10^{-2}	4.09x10^{-2}	-2.22
	Tpi1	2.98x10^{-2}	4.09x10^{-2}	-4.61
	Cycs	3.06x10^{-2}	4.09x10^{-2}	-3.63
	Ndufs2	3.16x10^{-2}	4.16x10^{-2}	-2.62
	Ugt2b10	3.58x10^{-2}	4.46x10^{-2}	1.85
	Cyp2c23	3.72x10^{-2}	4.52x10^{-2}	-1.61
	Csad	3.79x10^{-2}	4.52x10^{-2}	-2.42
	Atp5po	3.79x10^{-2}	4.52x10^{-2}	-3.22
	Maob	4.04x10^{-2}	4.56x10^{-2}	-2.63
	Pcyt1a	4.05x10^{-2}	4.56x10^{-2}	-2.9

	Chdh	4.15x10^{-2}	4.58x10^{-2}	-2.45
	Baat	5.00x10^{-2}	5.50x10^{-2}	-2.56
**Steroid hormone biosynthesis**	*Cyp2b1*	7.02x10^{-4}	1.21x10^{-2}	6.98
	Cyp2c13	3.67x10^{-3}	1.88x10^{-2}	-3.8
	Ugt2b1	4.77x10^{-3}	1.95x10^{-2}	2.99
	Sts	8.01x10^{-3}	2.41x10^{-2}	1.92
	Cyp3a2	1.43x10^{-2}	3.00x10^{-2}	-4.03
	Comt	2.05x10^{-2}	3.47x10^{-2}	-4.3
	Ugt2b10	3.58x10^{-2}	4.46x10^{-2}	1.85
	Cyp2c23	3.72x10^{-2}	4.52x10^{-2}	-1.61
**Steroid biosynthesis**	*Tm7sf2*	4.15x10^{-4}	9.54x10^{-3}	3.51
	Sqle	5.83x10^{-3}	2.14x10^{-2}	2.96
	Nsdhl	1.66x10^{-2}	3.13x10^{-2}	2.29
	Dhcr7	2.38x10^{-2}	3.67x10^{-2}	2.22
**Carbon metabolism**	*Rgn*	4.99x10^{-4}	1.08x10^{-2}	-4.46
	Esd	3.41x10^{-3}	1.88x10^{-2}	3.45
	Pc	5.62x10^{-3}	2.09x10^{-2}	-2.12
	Eno1	9.33x10^{-3}	2.54x10^{-2}	-3.43
	Eno3	1.31x10^{-2}	2.83x10^{-2}	2.8
	Aco2	1.55x10^{-2}	3.12x10^{-2}	-2.93
	Tpi1	2.98x10^{-2}	4.09x10^{-2}	-4.61
**Drug metabolism - cytochrome P450**	*Ugt2b1*	4.77x10^{-3}	1.95x10^{-2}	2.99
	Fmo1	1.84x10^{-2}	3.33x10^{-2}	-3.95
	Gsta3	3.35x10^{-2}	4.30x10^{-2}	-2.12
	Ugt2b10	3.58x10^{-2}	4.46x10^{-2}	1.85
	Maob	4.04x10^{-2}	4.56x10^{-2}	-2.63
**Ascorbate and aldarate metabolism**	*Rgn*	4.99x10^{-4}	1.08x10^{-2}	-4.46
	Ugt2b1	4.77x10^{-3}	1.95x10^{-2}	2.99
	Ugt2b10	3.58x10^{-2}	4.46x10^{-2}	1.85
**Biosynthesis of amino acids**	*Gpc4*	1.14x10^{-2}	2.71x10^{-2}	-2.52
	Eno1	9.33x10^{-3}	2.54x10^{-2}	-3.43
	Eno3	1.31x10^{-2}	2.83x10^{-2}	2.8
	Aco2	1.55x10^{-2}	3.12x10^{-2}	-2.93
	Tpi1	2.98x10^{-2}	4.09x10^{-2}	-4.61
**Taurine and hypotaurine metabolism**	*Csad*	3.79x10^{-2}	4.52x10^{-2}	-2.42
	Baat	5.00x10^{-2}	5.50x10^{-2}	-2.56
**Starch and sucrose metabolism**	*Enpp3*	4.50x10^{-3}	1.91x10^{-2}	-2.11
	Ugt2b1	4.77x10^{-3}	1.95x10^{-2}	2.99
	Ugt2b10	3.58x10^{-2}	4.46x10^{-2}	1.85

**Pentose and glucuronate interconversions**	*Sord*	2.27×10^{-3}	1.73×10^{-2}	-2.56
	Ugt2b1	4.77×10^{-3}	1.95×10^{-2}	2.99
	Ugt2b10	3.58×10^{-2}	4.46×10^{-2}	1.85
**Fructose and mannose metabolism**	*Sord*	2.27×10^{-3}	1.73×10^{-2}	-2.56
	Khk	2.65×10^{-3}	1.74×10^{-2}	-7.18
	Tpi1	2.98×10^{-2}	4.09×10^{-2}	-4.61
**Tyrosine metabolism**	*Fah*	4.05×10^{-4}	9.54×10^{-3}	-1.77
	Comt	2.05×10^{-2}	3.47×10^{-2}	-4.3
	Maob	4.04×10^{-2}	4.56×10^{-2}	-2.63
**Linoleic acid metabolism**	*Cyp2c13*	3.67×10^{-3}	1.88×10^{-2}	-3.8
	Cyp3a2	1.43×10^{-2}	3.00×10^{-2}	-4.03
	Cyp2c23	3.72×10^{-2}	4.52×10^{-2}	-1.61
**Arachidonic acid metabolism**	*Cyp2b1*	7.02×10^{-4}	1.21×10^{-2}	6.98
	Cyp2c13	3.67×10^{-3}	1.88×10^{-2}	-3.8
	Cyp4a10	1.66×10^{-2}	3.13×10^{-2}	2.2
	Cyp2c23	3.72×10^{-2}	4.52×10^{-2}	-1.61
**Histidine metabolism**	*Uroc1*	3.23×10^{-3}	1.88×10^{-2}	-5.39
	Maob	4.04×10^{-2}	4.56×10^{-2}	-2.63
**Glycolysis / Gluconeogenesis**	*Eno1*	9.33×10^{-3}	2.54×10^{-2}	-3.43
	Eno3	1.31×10^{-2}	2.83×10^{-2}	2.8
	Tpi1	2.98×10^{-2}	4.09×10^{-2}	-4.61

[1]*p*-value - Student's t-test *p*-value
[2]FDR *p*-value – FDR corrected *p*-value using the Benjamini-Hochberg method
[3]F.C. - fold change calculated using the average of integrated peak area from EtOH-feed samples divided by the average of the integrated peak area from control samples.

Table S5. Enriched pathway analysis of proteomics dataset using WikiPathways.

Pathway name	Wikipathway ID	Gene	*p*-value[1]	FDR *p*-value[3]	F.C.[3]
**Electron transport chain**	WP59	*Atp5po*	$3.79x10^{-2}$	$4.52x10^{-2}$	-3.22
		Ndufa6	$6.60x10^{-3}$	$2.28x10^{-2}$	-3.88
		Ndufs3	$1.62x10^{-2}$	$3.13x10^{-2}$	-2.01
		Cox6c2	$1.00x10^{-3}$	$1.34x10^{-2}$	-2.6
		Ndufs1	$8.90x10^{-3}$	$2.54x10^{-2}$	-3.3
		Ndufv2	$1.08x10^{-3}$	$1.34x10^{-2}$	-4.76
		Ndufb10	$3.70x10^{-3}$	$1.88x10^{-2}$	-7.17
		Atp1a2	$4.64x10^{-2}$	$4.83x10^{-2}$	-2.57
		Ndufa10	$7.80x10^{-3}$	$2.41x10^{-2}$	-2.67
		Ndufa2	$1.37x10^{-2}$	$2.92x10^{-2}$	-8.32
		Cox4i1	$2.49x10^{-3}$	$1.73x10^{-2}$	-3.09
		Cox5a	$4.44x10^{-3}$	$1.91x10^{-2}$	-3.6
		Atp5f1d	$4.21x10^{-3}$	$1.91x10^{-2}$	-22.63
		Mtnd4	$2.22x10^{-2}$	$3.60x10^{-2}$	-4.77
		Ndufs2	$3.16x10^{-2}$	$4.16x10^{-2}$	-2.62
		Cox7a2l	$4.35x10^{-3}$	$1.91x10^{-2}$	-11.5
**Oxidative phosphorylation**	WP1283	*Atp5po*	$3.79x10^{-2}$	$4.52x10^{-2}$	-3.22
		Ndufa6	$6.60x10^{-3}$	$2.28x10^{-2}$	-3.88
		Ndufs3	$1.62x10^{-2}$	$3.13x10^{-2}$	-2.01
		Ndufs1	$8.90x10^{-3}$	$2.54x10^{-2}$	-3.3
		Ndufv2	$1.08x10^{-3}$	$1.34x10^{-2}$	-4.76
		Ndufb10	$3.70x10^{-3}$	$1.88x10^{-2}$	-7.17
		Atp5f1d	$4.21x10^{-3}$	$1.91x10^{-2}$	-22.63
		Ndufa10	$7.80x10^{-3}$	$2.41x10^{-2}$	-2.67
		Ndufa2	$1.37x10^{-2}$	$2.92x10^{-2}$	-8.32
		Atp5mf	$1.35x10^{-3}$	$1.46x10^{-2}$	-1.83

		Mtnd4	2.22×10^{-2}	3.60×10^{-2}	-4.77
		Ndufs2	3.16×10^{-2}	4.16×10^{-2}	-2.62
**Glucuronidation**	WP1276	*Ugt2b1*	4.77×10^{-3}	1.95×10^{-2}	2.99
		Ugt2b10	3.58×10^{-2}	4.46×10^{-2}	1.85
		Ugdh	3.02×10^{-2}	4.09×10^{-2}	-2.59
		Ugp2	2.15×10^{-3}	1.69×10^{-2}	-2.54
**Nuclear receptors in lipid metabolism and toxicity**	WP139	*Cyp2e1*	2.78×10^{-6}	1.02×10^{-3}	8.95
		Cyp2c11	1.17×10^{-2}	2.71×10^{-2}	-3.75
		Abcb4	3.03×10^{-2}	4.09×10^{-2}	-2.55
		Abcb11	1.90×10^{-2}	3.37×10^{-2}	-2.12
		Abcc2	6.13×10^{-4}	1.13×10^{-2}	-4.51
**Hexoses metabolism in proximal tubules**	WP3916	*Eno3*	1.31×10^{-2}	2.83×10^{-2}	2.8
		Pdhb	3.73×10^{-3}	1.88×10^{-2}	-2.39
		Sord	2.27×10^{-3}	1.73×10^{-2}	-2.56
		Eno1	9.33×10^{-3}	2.54×10^{-2}	-3.43
		Pc	5.62×10^{-3}	2.09×10^{-2}	-2.12
		Khk	2.65×10^{-3}	1.74×10^{-2}	-7.18
**Cholesterol metabolism**	WP632	*Sqle*	5.83×10^{-3}	2.14×10^{-2}	2.96
		Nsdhl	1.66×10^{-2}	3.13×10^{-2}	2.29
		Dhcr7	2.38×10^{-2}	3.67×10^{-2}	2.22
		Scarb1	3.69×10^{-2}	4.52×10^{-2}	-2.94

[1]*p*-value - Student's t-test *p*-value
[2]FDR *p*-value – FDR corrected *p*-value using the Benjamini-Hochberg method
[3]F.C. - fold change calculated using the average of integrated peak area from EtOH-feed samples divided by the average of the integrated peak area from control samples.

References

1. Simpson, A.J.; Brown, S.A. Purge NMR: effective and easy solvent suppression. *J Magn Reson* **2005**, *175*, 340-346, doi:10.1016/j.jmr.2005.05.008.
2. Worley, B.; Powers, R. MVAPACK: a complete data handling package for NMR metabolomics. *ACS Chem Biol* **2014**, *9*, 1138-1144, doi:10.1021/cb4008937.
3. Pang, Z.; Chong, J.; Zhou, G.; de Lima Morais, D.A.; Chang, L.; Barrette, M.; Gauthier, C.; Jacques, P.E.; Li, S.; Xia, J. MetaboAnalyst 5.0: narrowing the gap between raw spectra and functional insights. *Nucleic Acids Res* **2021**, *49*, W388-W396, doi:10.1093/nar/gkab382.
4. Geva, S.; Sitte, J. Adaptive nearest neighbor pattern classification. *IEEE Trans Neural Netw* **1991**, *2*, 318-322, doi:10.1109/72.80344.
5. Perperoglou, A.; Sauerbrei, W.; Abrahamowicz, M.; Schmid, M. A review of spline function procedures in R. *BMC Med Res Methodol* **2019**, *19*, 46, doi:10.1186/s12874-019-0666-3.
6. Emwas, A.H.; Saccenti, E.; Gao, X.; McKay, R.T.; Dos Santos, V.; Roy, R.; Wishart, D.S. Recommended strategies for spectral processing and post-processing of 1D (1)H-NMR data of biofluids with a particular focus on urine. *Metabolomics* **2018**, *14*, 31, doi:10.1007/s11306-018-1321-4.
7. Luan, H.; Ji, F.; Chen, Y.; Cai, Z. statTarget: A streamlined tool for signal drift correction and interpretations of quantitative mass spectrometry-based omics data. *Anal Chim Acta* **2018**, *1036*, 66-72, doi:10.1016/j.aca.2018.08.002.

8. Yang, Q.; Wang, Y.; Zhang, Y.; Li, F.; Xia, W.; Zhou, Y.; Qiu, Y.; Li, H.; Zhu, F. NOREVA: enhanced normalization and evaluation of time-course and multi-class metabolomic data. *Nucleic Acids Res* **2020**, *48*, W436-W448, doi:10.1093/nar/gkaa258.
9. Wishart, D.S.; Feunang, Y.D.; Marcu, A.; Guo, A.C.; Liang, K.; Vazquez-Fresno, R.; Sajed, T.; Johnson, D.; Li, C.; Karu, N.; et al. HMDB 4.0: the human metabolome database for 2018. *Nucleic Acids Res* **2018**, *46*, D608-D617, doi:10.1093/nar/gkx1089.
10. Wishart, D.S.; Tzur, D.; Knox, C.; Eisner, R.; Guo, A.C.; Young, N.; Cheng, D.; Jewell, K.; Arndt, D.; Sawhney, S.; et al. HMDB: the Human Metabolome Database. *Nucleic Acids Res* **2007**, *35*, D521-526, doi:10.1093/nar/gkl923.
11. Crook, A.; De Lima Leite, A.; Payne, T.; Bhinderwala, F.; Woods, J.; Singh, V.K.; Powers, R. Radiation exposure induces cross-species temporal metabolic changes that are mitigated in mice by amifostine. *Sci Rep* **2021**, *11*, 14004, doi:10.1038/s41598-021-93401-7.
12. Chatterjee, A.; Sakallioglu, I.T.; Murthy, D.; Kosmacek, E.A.; Singh, P.K.; McDonald, J.T.; Powers, R.; Oberley-Deegan, R.E. MnTE-2-PyP protects fibroblast mitochondria from hyperglycemia and radiation exposure. *Redox Biol* **2022**, *52*, 102301, doi:10.1016/j.redox.2022.102301.
13. Xia, J.; Sinelnikov, I.V.; Han, B.; Wishart, D.S. MetaboAnalyst 3.0--making metabolomics more meaningful. *Nucleic Acids Res* **2015**, *43*, W251-257, doi:10.1093/nar/gkv380.
14. Bindea, G.; Mlecnik, B.; Hackl, H.; Charoentong, P.; Tosolini, M.; Kirilovsky, A.; Fridman, W.H.; Pages, F.; Trajanoski, Z.; Galon, J. ClueGO: a Cytoscape plug-in to

decipher functionally grouped gene ontology and pathway annotation networks. *Bioinformatics* **2009**, *25*, 1091-1093, doi:10.1093/bioinformatics/btp101.

15. Shannon, P.; Markiel, A.; Ozier, O.; Baliga, N.S.; Wang, J.T.; Ramage, D.; Amin, N.; Schwikowski, B.; Ideker, T. Cytoscape: a software environment for integrated models of biomolecular interaction networks. *Genome research* **2003**, *13*, 2498-2504, doi:10.1101/gr.1239303.

16. Kutsukake, T.; Furukawa, Y.; Ondo, K.; Gotoh, S.; Fukami, T.; Nakajima, M. Quantitative Analysis of UDP-Glucuronosyltransferase Ugt1a and Ugt2b mRNA Expression in the Rat Liver and Small Intestine: Sex and Strain Differences. *Drug Metab Dispos* **2019**, *47*, 38-44, doi:10.1124/dmd.118.083287.
